# Preparation, Characterization, and Application of pH-Response Color-Changeable Films Based on Pullulan, Cooked Amaranth (*Amaranthus tricolor* L.) Juice, and Bergamot Essential Oil

**DOI:** 10.3390/foods12142779

**Published:** 2023-07-21

**Authors:** Fengfeng Xu, Dawei Yun, Xiaoqian Huang, Bixue Sun, Chao Tang, Jun Liu

**Affiliations:** College of Food Science and Engineering, Yangzhou University, Yangzhou 225127, China; 15861329187@163.com (F.X.); daweiyun2021@126.com (D.Y.); hhhhhxq321@163.com (X.H.); aasun99@163.com (B.S.); 008155@yzu.edu.cn (C.T.)

**Keywords:** pullulan, essential oil, cooked amaranth juice, betacyanins, smart packaging

## Abstract

Pullulan-based smart packaging films were prepared by mixing cooked amaranth juice and bergamot essential oil. The impact of cooked amaranth juice and bergamot essential oil on the color-changeability, structural characterization, and barrier, antioxidant, mechanical and thermal properties of pullulan-based films was determined. Results showed the cooked amaranth juice contained pH-response color-changing betacyanins. The pullulan films containing cooked amaranth juice were color-changeable in pH 9–12 buffers and in ammonia vapor. The color-changeable property of betacyanins in cooked amaranth juice was unaffected by bergamot essential oils. The inner structure of pullulan films was greatly affected by cooked amaranth juice, forming big and ordered humps in film cross-sections. The crystallinity of pullulan films was improved by the combined addition of cooked amaranth juice and bergamot essential oil. Among the films, the pullulan film containing cooked amaranth juice and 6% bergamot essential oil showed the highest UV-vis light barrier property, antioxidant activity, and tensile strength; while the pullulan film containing cooked amaranth juice and 4% bergamot essential oil showed the highest oxygen barrier property and thermal stability. Moreover, the pullulan films containing cooked amaranth juice were able to monitor the freshness of shrimp by presenting color changes from reddish purple to dark red.

## 1. Introduction

The preparation of green and biodegradable food packaging films based on natural biopolymers (e.g., polysaccharides, lipids and proteins) has become a hot spot in the food industry [1,2]. Biopolymers are abundant, inexpensive, non-toxic and non-hazardous substances with high biodegradability and biocompatibility, providing a new solution to alleviate the serious environmental problems caused by using traditional plastic packaging [3]. Among various biopolymers, pullulan has received increasing attention because of its colorless, odorless, and good film-forming properties [4]. Pullulan-based packaging films are demonstrated to possess low permeability to oil and oxygen [5]. However, the application of the neat pullulan films is restricted by their brittleness, hydrophilicity, and lack of active ingredients [5]. In this regard, researchers have designed several strategies to improve the properties of pullulan films, such as using physical/chemical cross-linking, adding plasticizers, blending with other biopolymers, and adding active ingredients [4]. Among different solutions, the incorporation of active ingredients (e.g., natural pigments and essential oils) into pullulan films is very promising [6,7].

In recent years, natural pigments with pH-response color-changeable properties have attracted attention in the field of smart packaging [8,9,10]. Betacyanins, one kind of natural pigment, are distributed in the flowers, leaves, fruits, and rhizomes of plant order *Caryophyllales* [11]. Previous studies have demonstrated that betacyanins extracted from different plants possess good pH-response color-changing properties [12,13]. Since the spoilage of protein-rich food is accompanied by pH variation, the color changes of betacyanins embedded in biopolymer-based packaging films can be used to indicate the spoilage/freshness of protein-rich food [11]. Notably, the pH-response color-changeable property of the biopolymer-based packaging films with betacyanins depends on the amount of betacyanins in the films [12,14], the plant source of betacyanins [13], and the character of the biopolymer-based film matrix [15]. However, the development of pullulan-based smart packaging films containing betacyanins has not yet been reported.

Vegetable amaranth (*Amaranthus tricolor* L.) is a leafy vegetable that is widely cultivated throughout tropical and temperate zones of Asia [16]. The leaves of vegetable amaranth are good sources of natural pigments, such as betacyanins, chlorophyll, and carotenoids [17]. Recently, some researchers have found that betacyanins extracted from vegetable amaranth were suitable to prepare pH-response color-changeable films [18,19]. However, the extraction of betacyanins from vegetable amaranth normally involves a high quantity of organic solvents (e.g., ethanol and methanol), which are environmentally disadvantageous and have a high cost [20]. Meanwhile, other pigments (i.e., chlorophyll and carotenoids) are normally co-extracted from vegetable amaranth along with betacyanins [19,21]. The presence of chlorophyll and carotenoids can greatly interfere with the color-changeable property of betacyanins. Thus, researchers often adopt complicated separation/purification procedures to obtain relatively pure betacyanins before preparing color-changeable films [18]. Considering chlorophyll and carotenoids are liposoluble pigments with low stability under high temperatures, we used a simple and green method in this study to isolate betacyanins by directly cooking vegetable amaranth in boiling water. This method effectively avoided the usage of organic solvents for betacyanin extraction and the complicated separation/purification procedures for betacyanins.

Therefore, in this study, we prepared pH-response color-changeable films by using pullulan as the matrix and the cooked amaranth juice (CAJ) as colorants. At the same time, bergamot essential oil (BEO) was added into the films, acting as an active ingredient to improve film performance. Previous studies have demonstrated that BEO can effectively enhance the barrier and antioxidant properties of biopolymer-based films [22,23]. Despite this, the impact of BEO on pullulan-based films is still unknown. On the whole, pH-response color-changeable films were prepared based on pullulan, CAJ, and different amounts of BEO. The aim of this study was to evaluate the impact of CAJ and BEO on the performance of pullulan-based films. The composite films were used as color-changeable indicators to monitor the freshness of shrimp.

## 2. Materials and Methods

### 2.1. Material and Reagents

Fresh vegetable amaranth (*A. tricolor* L.) and shrimp (*Fenneropenaeus chinensis*) were purchased from the local Yonghui Superstore (Yangzhou, China). Pullulan was bought in Kangnaxin Biotechnology Co., Ltd. (Weifang, China). BEO was supplied by Huashuo Perfume Oil Co., Ltd. (Ji’an, China). Ethanol, glycerol, and 2,2-diphenyl-1-picrylhydrazyl (DPPH) were acquired from Sinopharm Chemical Reagent Co., Ltd. (Shanghai, China).

### 2.2. Preparation of Cooked Amaranth Juice (CAJ)

Fresh red amaranth (1000 g) was cooked in a stainless steel pot by boiling it in 1.5 L of distilled water at 100 °C for 15 min. The CAJ was collected by filtration and then concentrated to 50 mL using a rotary evaporator at 50 °C. The obtained concentrate was filtered through a 0.2 μm microporous membrane to remove insoluble substances. The red-colored filtrate was collected and stored at 4 °C in darkness before use. The total betacyanin content in the CAJ filtrate was determined as 1.875 mg/mL, following the method of Qin et al. [12]. The pH-response color-changeable property of the filtrate was checked by mixing 0.1 mL CAJ filtrate with 4 mL of a pH 3−12 solution and scanning the visible absorption spectrum of the mixture at 450−650 nm [24].

### 2.3. Preparation of Films

Different pullulan-based film-forming solutions with different amounts of BEO and CAJ were prepared based on the formulas presented in Table 1. First, the blank pullulan-based film-forming solution was prepared by dissolving 6.8 g of pullulan in 140 mL of hot water (95 °C) for 2 h, which was followed by the addition of 1.02 g of glycerol as the plasticizer. Then, pullulan-based film-forming solutions with BEO were prepared by mixing the pullulan/glycerol solution with different amounts (2%, 4%, and 6%) of BEO emulsions based on pullulan weight at 20 °C for 30 min. The BEO emulsions were obtained by homogenizing BEO and Tween 80 (mass ratio of 5:1) at 10,000 rpm for 4 min beforehand. Simultaneously, a pullulan-based film-forming solution with CAJ was prepared by mixing the pullulan/glycerol solution with 1 mL of CAJ filtrate at 20 °C for 30 min. Finally, pullulan-based film-forming solutions with CAJ and BEO were prepared by mixing the pullulan/glycerol solution with 1 mL of CAJ filtrate and different amounts (2%, 4%, and 6%) of BEO emulsions based on pullulan weight at 20 °C for 30 min. All film-forming solutions were poured into Plexiglass molds (25 cm × 25 cm) and dried at 30 °C. The nominate of the films was based on the formula of the film-forming solutions (Table 1), namely PL film (the blank pullulan film), PL-BEO2, PL-BEO4, and PL-BEO6 films (pullulan films with 2%, 4%, and 6% BEO emulsions), PL-CAJ film (pullulan film with CAJ), and PL-CAJ-BEO2, PL-CAJ-BEO4, and PL-CAJ-BEO6 films (pullulan films with CAJ and 2%, 4%, and 6% BEO emulsions). Before testing, all films were stabilized in a glass desiccator with 50% relative humidity (RH) at 20 °C for 48 h.

### 2.4. Characterization of Films

#### 2.4.1. Color-Related Properties

The films were photographed by placing rectangular film samples on ginkgo leaves. The color values (L*, a*, b* and Δ*E*) of film samples were assessed with a SC-80C colorimeter (Kangguang Corp., Beijing, China) under illuminant D65.

The color-changeable property of films was determined by exposing film samples to pH 3–12 solutions for 60 s, and to an ammonia atmosphere (generated from a 0.25 mol/L ammonia solution) for 5–300 min [25].

The storage stability of films was evaluated by storing film samples at 4 °C or 20 °C for 30 days. The Δ*E* changes of film samples were determined every 3 days using a colorimeter to reflect the color changes of films during storage [26].

#### 2.4.2. Structural Characterization

The microstructures of gold-sputtered film cross-sections were characterized by a Gemini SEM 300 (Carl Zeiss, Oberkochen, Germany). The XRD patterns of films were recorded by an AXS D8 Adv diffractometer (Bruker Inc., Karlsruhe, Germany) at a diffraction angle (2*θ*) of 5–75° with Cu-Kα radiation. The FT-IR spectra of films were collected via a Varian 670 FT-IR spectroscope (Agilent, Santa Clara, CA, USA) equipped with a horizontal attenuated total reflectance. The scanning range, resolution, and scanning time were 400–4000 cm^−1^, 4 cm^−1^, and 32 times, respectively.

#### 2.4.3. Barrier Properties

The barrier property of films against UV-vis light was determined by scanning film samples in a quartz cuvette of a UV/vis spectrophotometer (Lambda 35, PerkinElmer Ltd., Waltham, MA, USA) from 200–800 nm.

The barrier property of films against water vapor, expressed as water vapor permeability (WVP), was determined by the gravimetric method at 20 °C [27]. In brief, glass cups (50 mL) containing thoroughly dried silica gels (40 g, 0% RH) were sealed by film samples. The film-sealed glass cups were then placed in a glass desiccator with distilled water (100% RH). The gravimetric increase of film-sealed glass cups was determined as a function of time.

The barrier property of films against oxygen gas, expressed as oxygen permeability (OP), was measured with a Basic 201 gas permeability tester (Labthink Corp., Jinan, China) at 23 °C and 50% RH. In brief, the film sample was placed between upper and lower chambers of the tester, which was then fully evacuated. Afterwards, the upper chamber of the tester was filled with oxygen gas while the lower chamber of the tester was monitored for its pressure change along with time [28].

#### 2.4.4. Antioxidant Activity

To measure the antioxidant activity of films, different amounts of film samples were dissolved in distilled water with the final concentration of 4–20 mg/mL. Then, 1 mL of each film solution was reacted with 3 mL of DPPH ethanol solution (0.1 mmol/L) at 20 °C for 30 min in the dark. The reaction product was measured for its absorbance at 517 nm [29].

#### 2.4.5. Mechanical Properties

Two mechanical parameters of films—tensile strength (TS) and elongation at break (EAB)—were analyzed with a STX200 texture analyzer (Yishite Corp., Xiamen, China). In brief, film strips (1 cm × 6 cm) were fixed on the analyzer and stretched at 4 mm/s [30].

#### 2.4.6. Thermal Properties

The thermal degradation behavior of films was determined via thermogravimetric analysis (TGA), using an HTG-1 thermogravimetric analyzer (Henven Corp., Beijing, China). In brief, film samples (approximately 2.5 mg) were heated from 40 °C to 700 °C at 10 °C/min under nitrogen flow [31].

### 2.5. Application of Films in Intelligent Packaging

The color-changeable films were used as intelligent packaging to monitor the quality of shrimp at 4 °C for 7 days [25]. Fresh shrimps (170 g) progressively decayed in a sealed fresh-keeping box, which was accompanied by the release of volatile basic compounds. The alkaline atmosphere could be sensed by film samples attached to the inner surface of the fresh-keeping box. The shrimps were taken out every 24 h and measured for their total volatile basic nitrogen (TVB-N) levels. At the same time, color changes of film samples were photographed.

### 2.6. Statistical Analysis

To evaluate the impact of BEO and CAJ on pullulan-based films, SPSS 13.0 software (SPSS Inc., Chicago, IL, USA) was used to analyze the experimental results by ANOVA and Duncan multiple range tests. Results were expressed as mean ± standard deviation (SD) with a significant difference level of *p* < 0.05.

## 3. Results and Discussion

### 3.1. Characterization of CAJ

**Figure 1:**
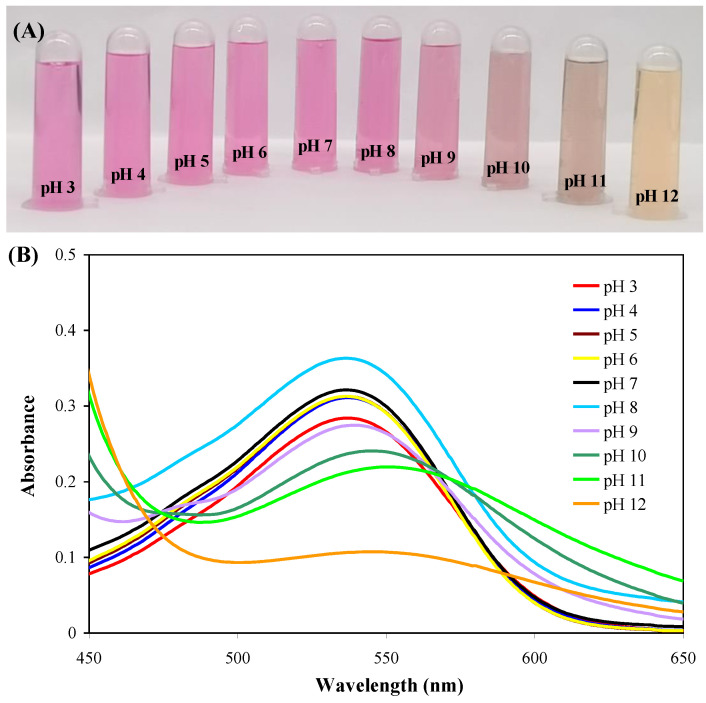
Color variations (**A**) and visible absorption spectra (**B**) of CAJ in different buffer solutions (pH 3–12).

The color and visible absorption spectrum of CAJ-buffer mixtures are shown in Figure 1. The CAJ-buffer mixtures showed a stable reddish purple color at pH 3–8 because betacyanins had stable structures in acidic/neutral solutions [18]. The UV-vis absorption spectrum of the CAJ-buffer mixtures showed the maximum absorption peak at 535 nm, corresponding to the characteristic absorption peak of betacyanins [13]. Meanwhile, the intensity of the maximum absorption peak increased with the increase of pH. The CAJ-buffer mixtures gradually faded and turned yellow at pH 9–12, which was caused by the aldimine bond hydrolysis of betacyanins, producing yellow-colored betalamic acid and colorless cyclo-dopa-5-*O*-(malonyl)-*β*-glucoside [24]. As a result, the maximum absorption peak of the CAJ-buffer mixtures significantly decreased at pH 9 and gradually shifted to higher wavelengths at pH 10–12. The color and UV-vis absorption spectrum of the CAJ-buffer mixtures were somewhat different from those of vegetable amaranth extract reported by other researchers [18,19]. Those researchers found that vegetable amaranth extract showed an olive color in alkaline solutions, which might be because the extract contained high amounts of chlorophyll and carotenoids [18,19]. In this study, chlorophyll and carotenoids were effectively removed by boiling water. Thus, the color changes of CAJ-buffer mixtures at pH 9–12 were mainly attributed to the structural decomposition of betacyanins.

### 3.2. Color-Related Properties of Films

**Figure 2:**
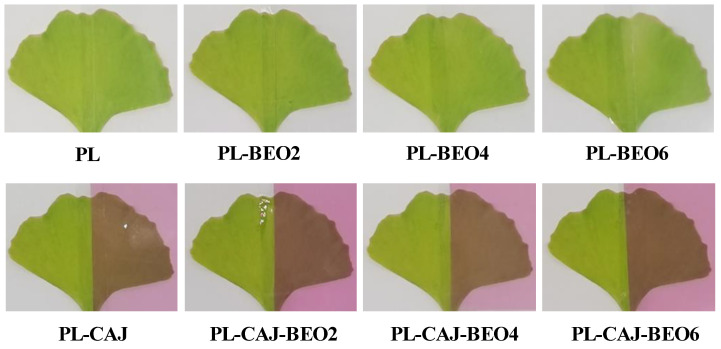
Initial color of PL, PL-BEO, PL-CAJ, and PL-CAJ-BEO films.

The color-changeable property is the most important function for smart packaging films. Figure 2 shows the color of pullulan films with different amounts of BEO and CAJ. The blank PL film and the three PL-BEO films were colorless, while PL-CAJ and the three PL-CAJ-BEO films were reddish purple. Yao et al. [13] also observed that starch/PVA films with betacyanins from different plants showed a reddish purple color. Table 2 shows that the color values of the films were unaffected by BEO, but were remarkably changed by CAJ. The films containing CAJ showed reduced L* and b* values, but increased a* and Δ*E* values. Similar results were observed by other researchers in the films containing betacyanins [12,13].

**Figure 3:**
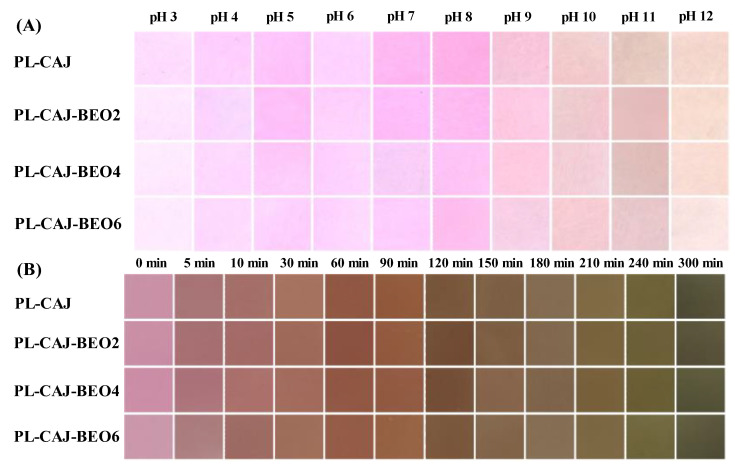
The pH-sensitivity (**A**) and ammonia-sensitivity (**B**) of PL-CAJ and PL-CAJ-BEO films.

Considering the color-changeable properties of CAJ (Figure 1A), we further tested the color-changeable properties of pullulan films containing CAJ (i.e., PL-CAJ film and three PL-CAJ-BEO films) in buffers and ammonia vapor. As shown in Figure 3, the films containing CAJ exhibited obvious color changes under alkaline environments, regardless of whether they were exposed to alkaline buffers or ammonia vapor. These results confirmed that the films containing CAJ had good color-changeable properties. Notably, no significant differences were noted between the PL-CAJ film and PL-CAJ-BEO films, indicating that BEO did not affect the color-changeable property of CAJ.

**Figure 4:**
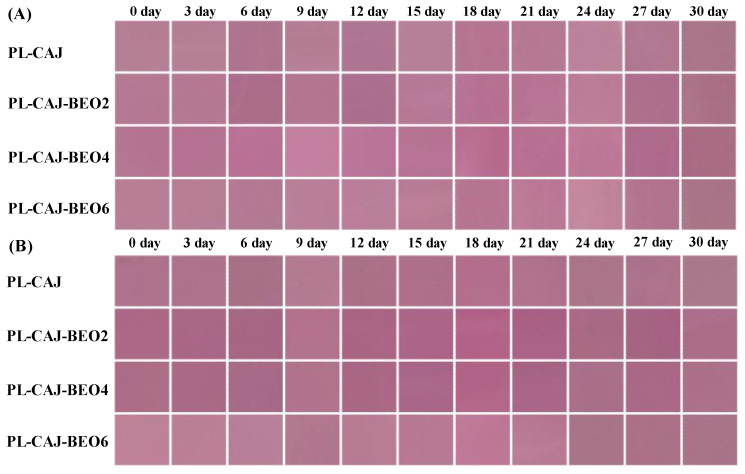
Color stability of PL-CAJ and PL-CAJ-BEO films stored at 4 °C (**A**) and 20 °C (**B**) for 30 days.

The storage stability of color-changeable films is highly needed in practical use. The pullulan films containing CAJ were observed for their color stability under 4 °C and 20 °C storage for 30 days. As shown in Figure 4, all the films presented the reddish purple color throughout the storage period, regardless of whether they were under 4 °C or 20 °C. Figure S1 showed that the Δ*E* value changes of all the films were smaller than 2, which could not be distinguished by the naked eye [15]. The above results suggested that pullulan films containing CAJ had good storage stability and could maintain the original reddish purple color during storage.

### 3.3. Structural Characterization of Films

**Figure 5:**
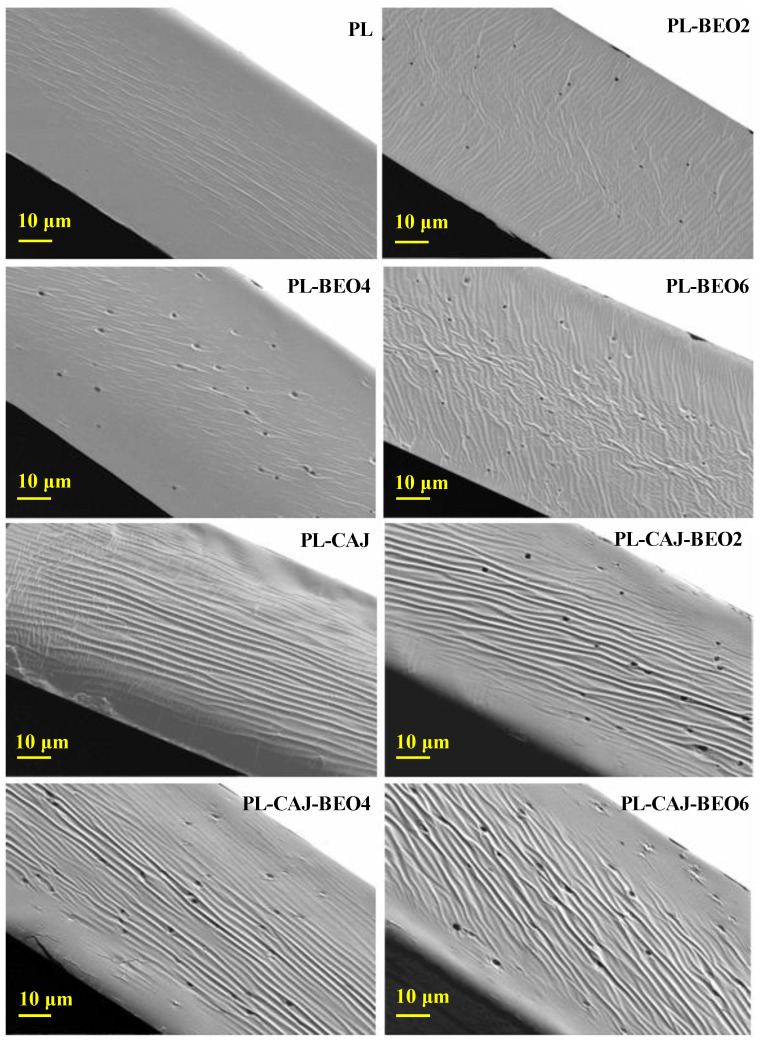
Cross-sectional morphologies of PL, PL-BEO, PL-CAJ, and PL-CAJ-BEO films.

The cross-sectional morphology of pullulan films with different amounts of BEO and CAJ was characterized by SEM (Figure 5). The blank PL film presented a continuous and intact cross-section with several ordered humps at the center of the film, indicating pullulan and glycerol were well mixed. Silva et al. [32] also observed that pullulan film plasticized with glycerol presented a uniform and continuous cross-sectional structure. Only the incorporation of 2%, 4%, and 6% BEO caused the formation of some micropores inside the films. The number of micropores increased with the increase of BEO amount. At the same time, the humps in the films became disordered and spread from the center to the side. Chu et al. [33] also found that pullulan films with cinnamon essential oil showed disordered cross-sections with micropores. Only the incorporation of CAJ caused the films to form some big and ordered humps. Hu et al. [18] also documented that vegetable amaranth extract made fish gelatin/quaternary ammonium chitosan films become wrinkled. The above results suggested that BEO and CAJ had different impacts on the inner structure of pullulan films. The simultaneous incorporation of BEO and CAJ had a duplicate effect on the films, where the micropores and big humps were simultaneously noted in PL-CAJ-BEO films.

**Figure 6:**
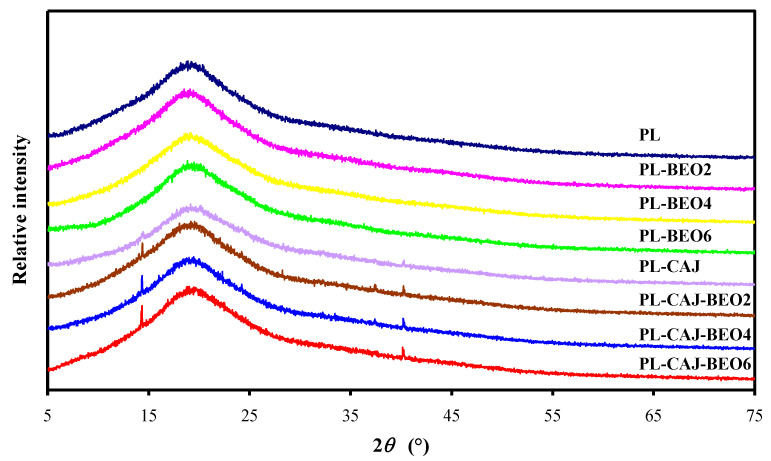
XRD patterns of PL, PL-BEO, PL-CAJ, and PL-CAJ-BEO films.

The XRD patterns of pullulan films with different amounts of BEO and CAJ are presented in Figure 6. Since pullulan was a kind of amorphous polysaccharide, the blank PL film showed a broad peak at 19°. A similar result was observed by Silva et al. [32] in pullulan film plasticized with glycerol. The incorporation of 2%, 4%, and 6% BEO emulsions only did not change the XRD patterns of films. Haghighatpanah et al. [34] also found that marjoram essential oil had no impact on the crystallinity of pullulan-based films. However, the incorporation of CAJ only made the film form some small sharp crystalline peaks at 14° and 40°, which were consistent with the big and ordered humps observed in the inner PL-CAJ film. This was because betacyanins in CAJ interacted with pullulan, increasing the ordered degree of the film. Three PL-CAJ-BEO films showed stronger crystalline peaks at 14° and 40°, compared to PL-CAJ film. It indicated that the ordered degree of PL-CAJ film was improved by BEO, due to the interactions between CAJ and BEO. Thus, it could be concluded that the simultaneous incorporation of BEO and CAJ had a synergistic effect on increasing the crystallinity of pullulan films.

**Figure 7:**
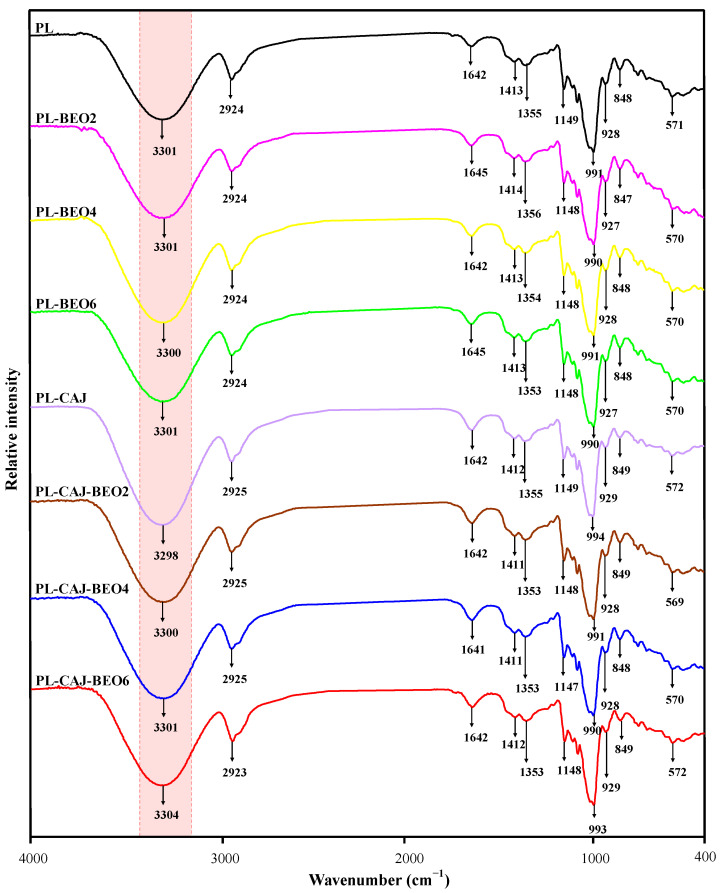
FT-IR spectra of PL, PL-BEO, PL-CAJ, and PL-CAJ-BEO films.

The interactions of film constituents were determined using FT-IR spectroscopy (Figure 7). The blank PL film showed the characteristic bands of pullulan at 3301, 2924, 1642, 1355, and 991–1149 cm^−1^, corresponding to O–H stretching, C–H stretching, bound water, O–H bending and α-D-glucopyranoside units, respectively [34,35]. Other films only showed minor differences compared to the blank PL film, which manifested in the O–H stretching region. The incorporation of BEO and CAJ made the intensity of O–H stretching increase to varying degrees, indicating the formation of hydrogen bonds between film constituents. Other researchers also observed that the O–H stretching band of polysaccharide-based films intensified after cinnamon essential oil and betacyanins were added [12,33]. Notably, the strongest O–H stretching was noted in PL-CAJ films, suggesting the hydrogen bonding interactions between betacyanins and pullulan were very strong. This was because both betacyanins and pullulan contained plenty of hydroxyl groups that could interact with each other through hydrogen bonds. However, the hydrogen bonding interactions between CAJ and pullulan were reduced by BEO due to the formation of CAJ and BEO interactions.

### 3.4. Barrier Property of Films

**Figure 8:**
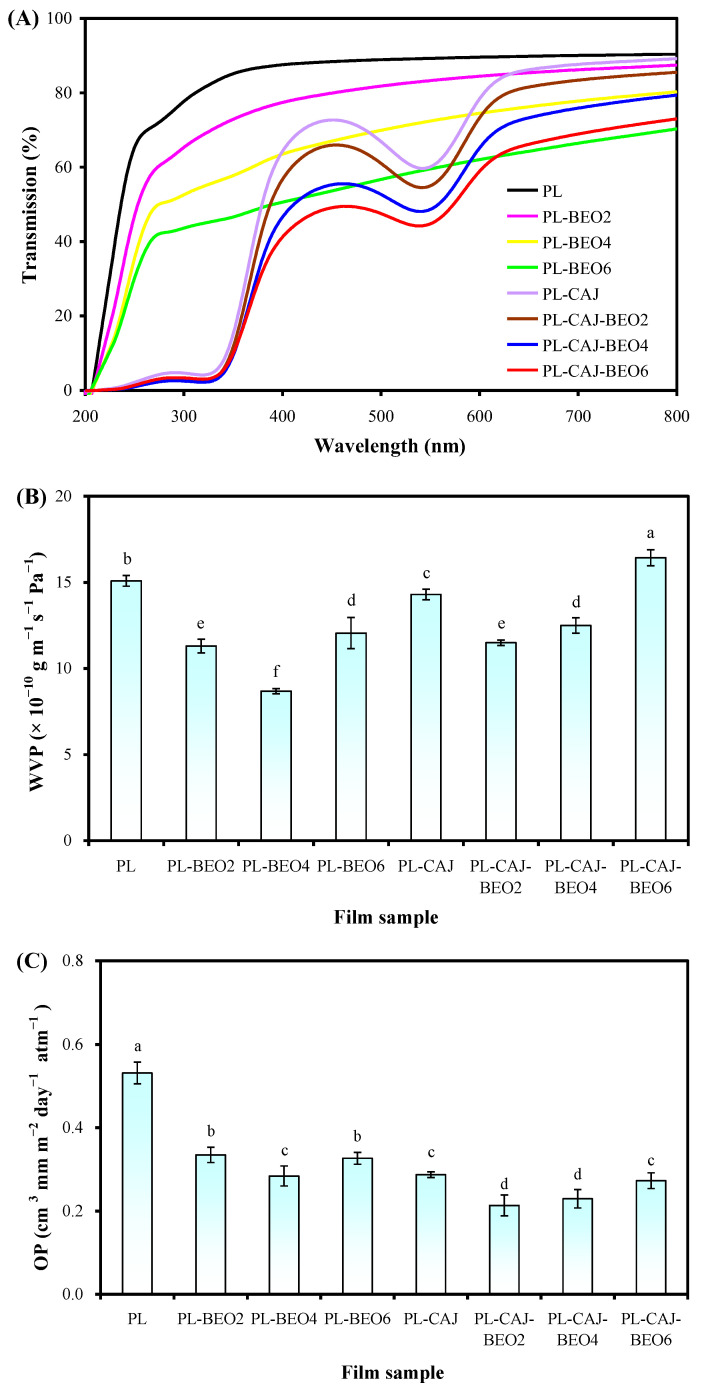
Light transmittance (**A**), WVP (**B**), and OP (**C**) of PL, PL-BEO, PL-CAJ, and PL-CAJ-BEO films. Each value represents mean ± SD (*n* = 3 for light transmittance and WVP, and *n* = 6 for OP). Different lowercase letters indicate a statistically significant difference (*p* < 0.05) among different films.

The light barrier properties of pullulan films with different amounts of BEO and CAJ are shown in Figure 8A. The UV-vis light transmittance of pullulan films was greatly elevated by BEO and CAJ in the UV and visible light ranges. The UV-vis light transmittance of the films containing BEO (i.e., PL-BEO film and three PL-CAJ-BEO films) gradually decreased with the increase of BEO amount, confirming the function of BEO in improving the light barrier property of pullulan-based films. The films containing CAJ (i.e., PL-CAJ film and three PL-CAJ-BEO films) showed superior UV light barrier properties because betacyanins contain several chromophores, such as C=N, C=O, and C=C [13]. Meanwhile, the PL-CAJ film and three PL-CAJ-BEO films showed the characteristic absorption band of betacyanins at 535 nm, revealing that the betacyanins in the films were very stable. However, the characteristic absorption band of betacyanins was not observed in the previously reported films containing vegetable amaranth extract [18,19], which was probably because vegetable amaranth extract contained high contents of chlorophyll and carotenoids. Therefore, our results suggest that the betacyanins obtained by boiling water treatment of vegetable amaranth are relatively pure. Notably, the simultaneous incorporation of BEO and CAJ had some synergistic effect on increasing the light barrier properties of pullulan films, with the PL-CAJ-BEO6 film having the highest light barrier property.

The water vapor barrier properties of pullulan films, with different amounts of BEO and CAJ, are shown in Figure 8B. The WVP of pullulan films decreased by 20.15−42.48% after solely BEO was added. The WVP of PL-BEO films depended on BEO amount, where a moderate BEO amount (4%) resulted in the lowest WVP. The WVP decrease in PL-BEO films was attributed to the hydrophobic character of essential oils [33]. Meanwhile, the emulsified BEO dispersed in the films could increase the curvature of the water molecule permeation path to a certain extent [36,37]. The PL-CAJ film showed a lower WVP than the PL film, but a higher WVP than PL-BEO films, suggesting that CAJ had a weaker impact on reducing the WVP of pullulan films compared to BEO. This was because betacyanins in CAJ were hydrophilic substances [24]. Yao et al. [13] also reported that betacyanins from different plant sources could only reduce the WVP of polysaccharide-based films to a limited degree. Notably, the simultaneous incorporation of BEO and CAJ had an antagonistic effect on reducing the WVP of pullulan films, with PL-CAJ-BEO6 film having a higher WVP than the blank PL film. This was mainly because the interactions between BEO and CAJ restricted their actions on the films.

The oxygen barrier properties of pullulan films with different amounts of BEO and CAJ are shown in Figure 8C. The OP of pullulan films was reduced by 37.74−47.17% after solely BEO was added. Among three PL-BEO films, the PL-BEO4 film showed the lowest OP. This result was consistent with WVP determination, where the PL-BEO4 film had the lowest WVP among the three PL-BEO films (Figure 6B). The reduced WVP in the films could be explained by the low oxygen solubility of BEO [38]. Acosta et al. [39] also found that when different kinds of essential oils were added, the OP of polysaccharide-based films decreased significantly. Notably, CAJ was very effective in reducing the OP of pullulan films. The PL-CAJ film showed comparable OP with the PL-BEO4 film. This was because the distribution of betacyanins in the films made the oxygen diffusion path become more complicated [40]. Other researchers also observed that the OP of polysaccharide-based films was significantly reduced by betacyanins from other plant sources [41,42]. Compared to the PL-BEO4 film and PL-CAJ film, the PL-CAJ-BEO2 film and PL-CAJ-BEO4 film showed a relatively lower OP, suggesting that the simultaneous incorporation of BEO and CAJ had a synergistic effect on reducing the OP of pullulan films.

### 3.5. Antioxidant Activity of Films

**Figure 9:**
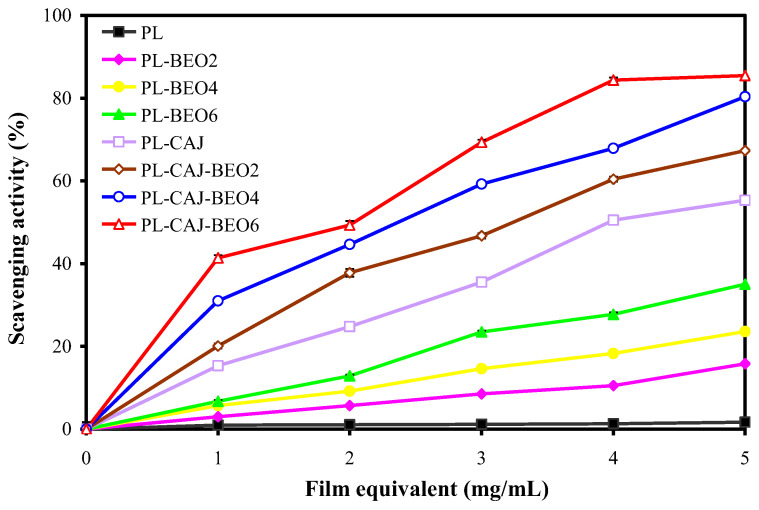
DPPH radical scavenging activity of PL, PL-BEO, PL-CAJ, and PL-CAJ-BEO films. Each value represents mean ± SD (*n* = 3).

Oxidation is one of the main causes of nutrient loss and spoilage of food. Antioxidant activity is an important requirement for food packaging films. The antioxidant activity of pullulan films was significantly elevated by adding different amounts of BEO and CAJ (Figure 9). The antioxidant activity of PL-BEO films increased with the BEO amount, which was because essential oils contained several antioxidant ingredients, such as terpenes, phenylpropanoids, aldehydes, esters, alcohols, and ketones [43]. The antioxidant activity of the PL-CAJ film was higher than that of PL-BEO films, attributed to the good free radical scavenging activity of betacyanins. The antioxidant activity of betacyanins was related to the hydrogen-atom-donating ability of phenolic hydroxyl groups [14]. Notably, the simultaneous incorporation of BEO and CAJ had a synergistic effect on increasing the antioxidant activity of pullulan films, with the PL-CAJ-BEO6 film having the highest antioxidant activity.

### 3.6. Mechanical Property of Films

**Figure 10:**
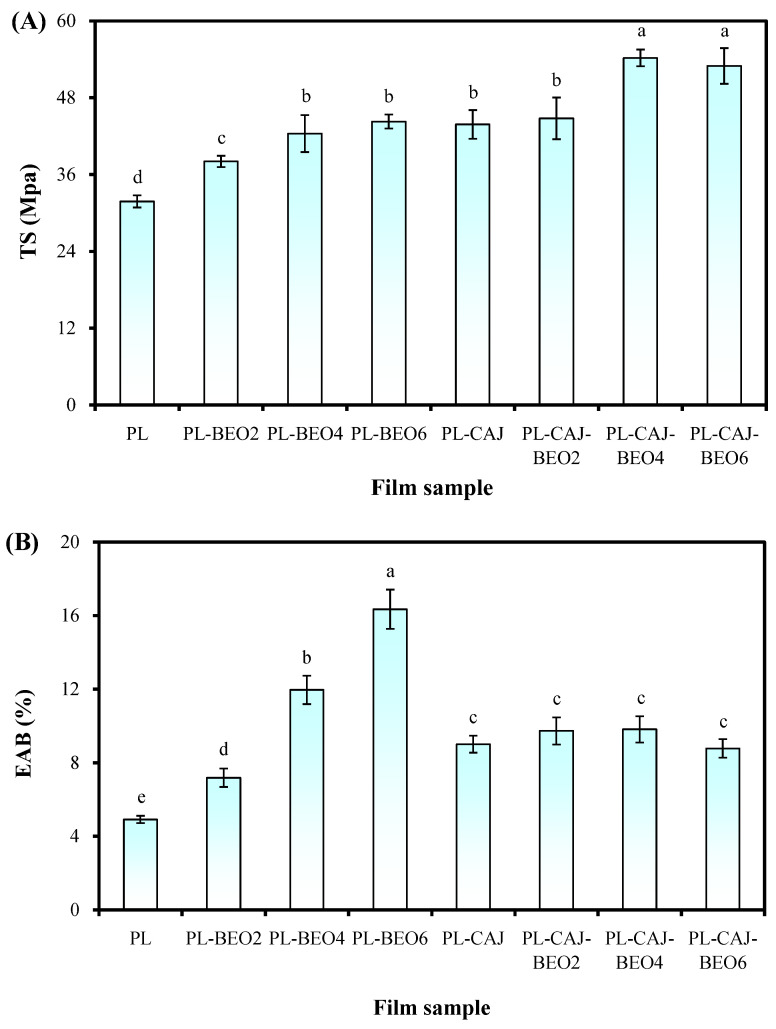
TS (**A**) and EAB (**B**) of PL, PL-BEO, PL-CAJ, and PL-CAJ-BEO films. Each value represents mean ± SD (*n* = 6 for TS and EAB). Different lower case letters indicate a statistically significant difference (*p* < 0.05) among different films.

The mechanical properties of packaging films, influenced by many factors (e.g., film constituents and film preparation conditions), are commonly reflected by TS and EAB. As shown in Figure 10A, the TS of pullulan films was increased by different amounts of BEO and CAJ. The BEO dispersed in the films formed interactions with film matrix and increased the TS of the films in an amount-dependent manner. Haghighatpanah et al. [34] suggested that essential oils could change the original arrangement of polysaccharide-based chains and limit film fluidity. In the study of Haghighatpanah et al. [34], the TS of mung bean protein isolate/pullulan films was improved using oregano essential oil. The PL-CAJ film showed a similar TS with PL-BEO4 and PL-BEO6 films, demonstrating that CAJ was very effective in elevating the TS of pullulan films. This was because betacyanins formed strong hydrogen bonding interactions with pullulan (Figure 7) and strengthened the entanglement of polysaccharide-based chains [19]. The TS of the PL-CAJ film was further elevated with 4% and 6% BEO, revealing the synergistic effect of BEO and CAJ on improving the TS of pullulan films. The above results were consistent with the SEM observation that films containing CAJ showed big and ordered humps inside the films (Figure 5), which might contribute to the TS enhancement of the films. Figure 10B showed that both BEO and CAJ had a big impact on improving the EAB of pullulan films. The EAB of pullulan films increased by between 46.29% and 232.95% after different amounts of BEO were added. With the increase of BEO amount, the EAB of pullulan films showed a sharp increasing trend. This was because BEO formed oil droplets in the films and made the film deform more easily [44]. Bu et al. [45] also reported that tea tree essential oil had a good plasticizing effect on konjac glucomannan/pullulan films. The EAB of pullulan films increased by 83.37% after CAJ was added, demonstrating that CAJ possessed a certain plasticizing effect. Similarly, other researchers documented that betacyanins could increase the EAB of polysaccharide-based films [12,13]. However, the further addition of BEO into the PL-CAJ film did not remarkably improve the EAB of the film. This was because CAJ and BEO interacted with each other and restricted their plasticizing effects. Thus, the results suggested that BEO and CAJ had an antagonistic effect on the EAB of pullulan films.

### 3.7. Thermal Property of Films

**Figure 11:**
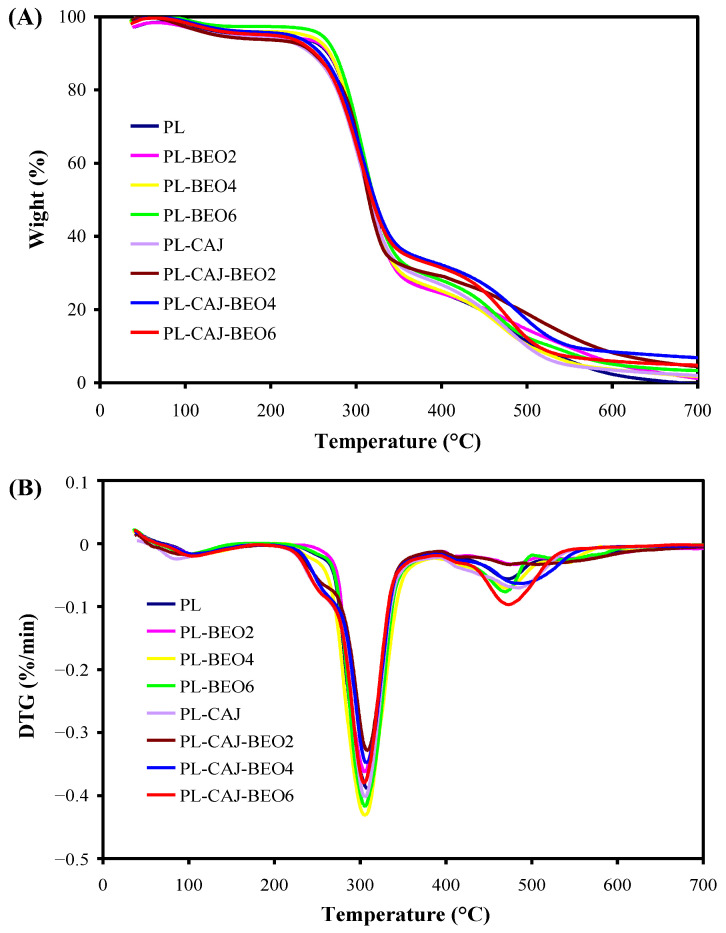
TGA (**A**) and DTG (**B**) curves of PL, PL-BEO, PL-CAJ, and PL-CAJ-BEO films.

The thermal properties of pullulan films with different amounts of BEO and CAJ is shown in Figure 11. The thermal degradation process of all the films contained three stages. The first stage (40–150 °C) was attributed to the evaporation of moisture [45]. The second stage (150–380 °C) was mainly caused by the degradation of glycerol, BEO, and betacyanins [34]. At this stage, the films containing CAJ (i.e., the PL-CAJ film and three PL-CAJ-BEO films) showed significant weight losses at 220–275 °C, which could be attributed to the decomposition of betacyanins. The rapidest weight loss rates of all the films were also noted at the second stage, locating at 307–311 °C. Yao et al. [46] also found that polysaccharide-based double-layer films containing betacyanins showed the rapidest weight loss rates at 311–317 °C. The third stage (380–700 °C) corresponded to the breakage and degradation of pullulan chains [47,48]. Notably, the blank PL film completely decomposed at 700 °C, whereas the rest of the films still contained some residual char at 700 °C. It indicated the thermal degradation process of pullulan films was retarded by BEO and CAJ. This result was consistent with the elevated TS in the films containing BEO and CAJ (Figure 10A). Among the films, the PL-CAJ-BEO4 film had the most residual char, suggesting BEO and CAJ had a synergistic effect on increasing the thermal stability of pullulan films.

### 3.8. Application of Films

Protein-rich shrimp can undergo enzymatic hydrolysis and protein decomposition during storage, producing a high quantity of organic amines and creating an alkaline atmosphere in the package [49]. The betacyanins in pH-response color-changeable films can react with organic amines and show intuitive color changes that can be easily observed by the naked eye. In this way, the pH-response color-changeable films can be used as freshness indicators to convey the quality information of shrimp to consumers and distributors by presenting visible color changes. Different from the conventionally used quantitative methods (e.g., TVB-N determination), the use of pH-response color-changeable films is a qualitative method that is more intuitive and convenient. Meanwhile, the use of pH-response color-changeable films does not require auxiliary equipment and can achieve real-time freshness information about shrimp [50]. Here, fresh shrimp was selected as the representative food to test the practical performance of color-changeable films. As shown in Table 3, the films containing CAJ (i.e., PL-CAJ and three PL-CAJ-BEO films) showed similar color-changing degrees, changing from an initial reddish purple color (day 0 to day 2) to a dark red color (day 3 to day 6) and finally, a brown color (day 7). Notably, the TVB-N level of shrimp exceeded the freshness threshold (20 mg/100 g) on day 3, which corresponded well with the dark red color of the films on day 3. The above results suggested that the freshness of shrimp could be simply distinguished by observing the color of the films containing CAJ. The presence of BEO did not interfere with the color-changeable properties of CAJ, which was consistent with the results of the pH-/ammonia-sensitive tests in Figure 3. In previous studies [18,19], researchers found that the films containing vegetable amaranth extract turned yellow when protein-rich food (fish, chicken and shrimp) became un-fresh. However, in the present study, the films containing CAJ did not turn yellow even on day 7. This was mainly because CAJ contained relatively pure betacyanins.

## 4. Conclusions

The pH-response color-changeable films were successfully prepared by using a pullulan matrix, betacyanin-rich CAJ, and different amounts of BEO. CAJ endowed the films with a reddish purple color and color changing properties. Both CAJ and BEO improved the UV-vis light, water vapor, oxygen barrier properties, antioxidant activity, tensile strength, elongation at break, and thermal stability of pullulan films. However, CAJ and BEO had different impacts on the structural characterization and barrier, antioxidant, and mechanical properties of pullulan films. CAJ and BEO had synergistic effects on increasing the crystallinity, light and oxygen barrier properties, antioxidant activity, TS, and thermal stability of pullulan films. The films containing CAJ were able to monitor shrimp freshness. Our results suggest that the prepared films could be used as smart packaging materials in the food industry.

## Figures and Tables

**Table 1:** Formula of each film-forming solution.

Films	Pullulan (g)	Glycerol (wt% on Pullulan)	CAJ (mL)	BEO Emulsions (wt% on Pullulan)	Distilled Water (mL)
PL	6.8	15	0	0	140
PL-BEO2	6.8	15	0	2	140
PL-BEO4	6.8	15	0	4	140
PL-BEO6	6.8	15	0	6	140
PL-CAJ	6.8	15	1	0	140
PL-CAJ-BEO2	6.8	15	1	2	140
PL-CAJ-BEO4	6.8	15	1	4	140
PL-CAJ-BEO6	6.8	15	1	6	140

**Table 2:** Color parameters including L*, a*, b*, and Δ*E* of PL, PL-BEO, PL-CAJ, and PL-CAJ-BEO films.

Films	L*	a*	b*	Δ*E*
PL	89.44 ± 0.03 ^a^	0.34 ± 0.01 ^d^	−2.94 ± 0.01 ^a^	4.98 ± 0.02 ^e^
PL-BEO2	89.46 ± 0.01 ^a^	0.26 ± 0.02 ^d^	−2.97 ± 0.01 ^ab^	4.98 ± 0.01 ^e^
PL-BEO4	89.52 ± 0.05 ^a^	0.33 ± 0.06 ^d^	−2.97 ± 0.01 ^ab^	4.94± 0.04 ^e^
PL-BEO6	89.53 ± 0.08 ^a^	0.37 ± 0.02 ^d^	−3.04 ± 0.04 ^b^	4.98 ± 0.04 ^e^
PL-CAJ	72.87 ± 0.07 ^b^	22.90 ± 0.06 ^c^	−10.04 ± 0.08 ^d^	32.56 ± 0.06 ^d^
PL-CAJ-BEO2	69.30 ± 0.03 ^e^	25.74 ± 0.07 ^a^	−10.73 ± 0.07 ^f^	38.49 ± 0.05 ^a^
PL-CAJ-BEO4	72.40 ± 0.22 ^c^	23.00 ± 0.20 ^c^	−9.68 ± 0.17 ^c^	32.83 ± 0.05 ^c^
PL-CAJ-BEO6	70.30 ± 0.01 ^d^	25.86 ± 0.08 ^b^	−10.40 ± 0.08 ^e^	36.42 ± 0.02 ^b^

Values are given as mean ± SD (*n* = 10). Different lower case letters in the same column indicate a significant difference (*p* < 0.05).

**Table 3:** TVB-N level changes of shrimp during cold storage for 7 days and the color changes of PL-CAJ and PL-CAJ-BEO films.

Time (day)	0	1	2	3	4	5	6	7
TVB-N level (mg/100 g)	4.68 ± 0.18 ^h^	9.07 ± 0.22 ^g^	14.85 ± 0.28 ^f^	21.53 ± 0.17 ^e^	26.11 ± 0.17 ^d^	35.24 ± 0.47 ^c^	44.57 ± 1.05 ^b^	57.51± 0.97 ^a^
PL-CAJ	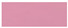	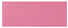	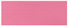	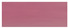	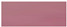	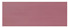	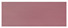	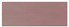
PL-CAJ-BEO2	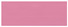	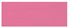	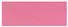	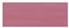	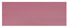	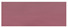	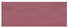	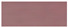
PL-CAJ-BEO4	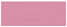	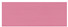	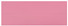	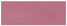	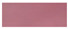	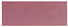	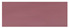	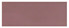
PL-CAJ-BEO6	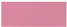	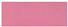	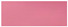	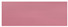	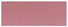	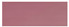	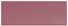	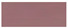

Values are given as mean ± SD (*n* = 3). Different lower case letters in the same column indicate significantly different (*p* < 0.05).

## Data Availability

The data used to support the findings of this study can be made available by the corresponding author upon request.

## Supplementary Materials

The following supporting information can be downloaded at: https://www.mdpi.com/article/10.3390/foods12142779/s1, Figure S1: Δ*E* value changes of PL-CAJ and PL-CAJ-BEO films stored at 4 °C (A) and 20 °C (B) for 30 days. Each value represents mean ± SD (*n* = 3).
