# Supplementary material for: Preparation, Characterization, and Application of pH-Response Color-Changeable Films Based on Pullulan, Cooked Amaranth (Amaranthus tricolor L.) Juice, and Bergamot Essential Oil
Source: Foods. 2023 Jul 21;12(14):2779. doi: 10.3390/foods12142779 (PMC10379735; doi:10.3390/foods12142779)
Supplement: Supplementary file 1 [file foods-12-02779-s001.zip › foods-2503259-supplementary.pdf]

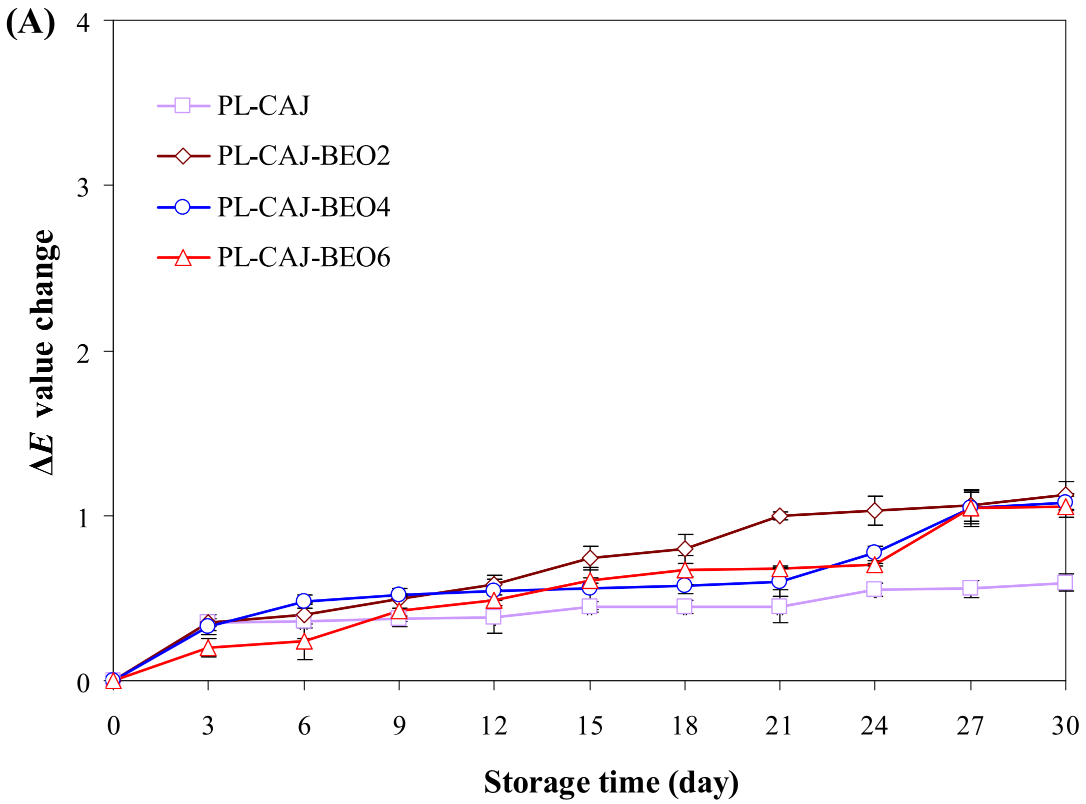

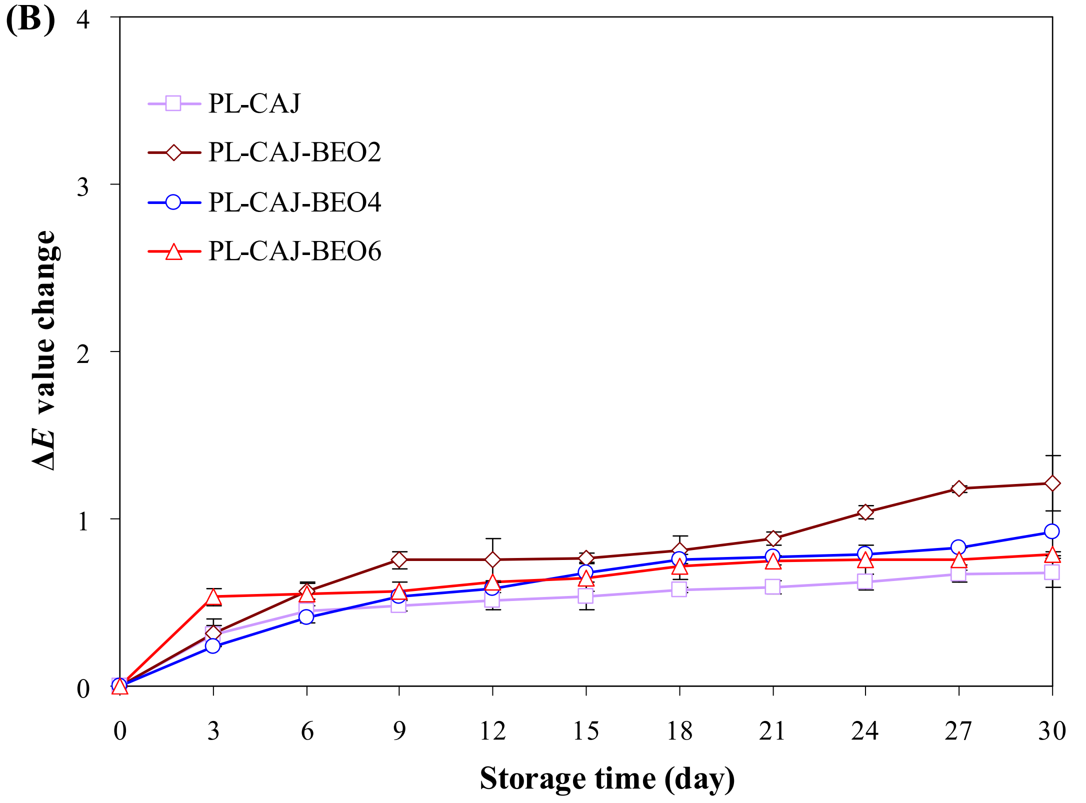

Figure S1. Δ*E* value changes of PL-CAJ and PL-CAJ-BEO films films stored at 4 °C (A) and 20 °C (B) for 30 days. Each value represents mean ± SD (n = 3).
